# Modification of a SERS-active Ag surface to promote adsorption of charged analytes: effect of Cu^2+^ ions

**DOI:** 10.3762/bjnano.12.67

**Published:** 2021-08-16

**Authors:** Bahdan V Ranishenka, Andrei Yu Panarin, Irina A Chelnokova, Sergei N Terekhov, Peter Mojzes, Vadim V Shmanai

**Affiliations:** 1 Institute of Physical Organic Chemistry, National Academy of Sciences of Belarus, 13 Surganova Str., Minsk, 220072, Belarus; 2 B. I. Stepanov Institute of Physics, National Academy of Sciences of Belarus, 68 Nezavisimosti Ave., 220072, Minsk, Belarus; 3 Institute of Radiobiology of NAS of Belarus, Feduninskogo st., 4, 246007, Gomel, Belarus; 4 Institute of Physics, Charles University, Ke Karlovu 5, CZ-121 16 Prague 2, Czech Republic

**Keywords:** electrostatic interaction, oligonucleotides, porphyrin, silver nanoparticles, substrate modification, surface-enhanced Raman spectroscopy (SERS)

## Abstract

This work studies the impact of the electrostatic interaction between analyte molecules and silver nanoparticles (Ag NPs) on the intensity of surface-enhanced Raman scattering (SERS). For this, we fabricated nanostructured plasmonic films by immobilization of Ag NPs on glass plates and functionalized them by a set of differently charged hydrophilic thiols (sodium 2-mercaptoethyl sulfonate, mercaptopropionic acid, 2-mercaptoethanol, 2-(dimethylamino)ethanethiol hydrochloride, and thiocholine) to vary the surface charge of the SERS substrate. We used two oppositely charged porphyrins, cationic copper(II) tetrakis(4-*N*-methylpyridyl) porphine (CuTMpyP4) and anionic copper(II) 5,10,15,20-tetrakis(4-sulfonatophenyl)porphine (CuTSPP4), with equal charge value and similar structure as model analytes to probe the SERS signal. Our results indicate that the SERS spectrum intensity strongly, up to complete signal disappearance, correlates with the surface charge of the substrate, which tends to be negative. Using the data obtained and our model SERS system, we analyzed the modification of the Ag surface by different reagents (lithium chloride, polyethylenimine, polyhexamethylene guanidine, and multicharged metal ions). Finally, all those surface modifications were tested using a negatively charged oligonucleotide labeled with Black Hole Quencher dye. Only the addition of copper ions into the analyte solution yielded a good SERS signal. Considering the strong interaction of copper ions with the oligonucleotide molecules, we suppose that inversion of the analyte charge played a key role in this case, instead of a change of charge of the substrate surface. Changing the charge of analytes could be a promising way to get clear SERS spectra of negatively charged molecules on Ag SERS-active supports.

## Introduction

Surface-enhanced Raman scattering (SERS) with its advantages of extreme sensitivity, high selectivity, and non-destructive nature has demonstrated great potential for the quick detection of chemicals in different samples [1]. It became popular in the scientific community during the last decades due to great prospects for practical solutions of, particularly, analytical problems [2]. However, despite the promising potential, it turned out that a lot of practical, theoretical, and even technical tasks need to be solved for practical applications of the method [2–4].

Raman signal surface enhancement uses so-called SERS-active substrates that are mainly inorganic or hybrid nanostructured materials. Significant attention has been devoted to the development of formation methods of metallic NP arrays with controllable parameters such as size, shape, interparticle distance, and ordering degree [5–12], with a focus on plasmonic structures with a high density of “hot spots”. Due to the progress in nanotechnology, a large number of highly sensitive SERS substrates has been synthesized [1,13].

The design of SERS substrates commonly aims at maximizing the plasmonic effect of Raman enhancement. There are two generally recognized mechanisms responsible for the SERS enhancement, namely electromagnetic enhancement (EM) and chemical enhancement (CE) [14–15]. The basic mechanism is EM through localized surface plasmon resonances (LSPRs) on the metal surface [16]. CE is at least two orders of magnitude weaker than EM. The CE mechanism is supposed to be caused by a charge transfer between the plasmonic surface and the chemically adsorbed analyte molecules, which introduces new states in the electronic structure of the metal–adsorbate complex leading to an increase in the Raman scattering cross section of the analyte [17]. Consequently, the CE mechanism should be accompanied by a change of spectral properties of the analyte, which was not observed in this study. Thus, we suppose EM is dominating in our work.

Since the LSPR-enhanced electromagnetic field decays exponentially with the distance from the metal surface, the analyte molecules should be located near the surface of the SERS substrate to achieve maximum enhancement. However, close proximity is not optimal because of possible quantum tunneling effects [2]. The SERS in the “hot spots” suffers from those undesired effects even more because the analyte molecules have to be localized in a small volume in gaps between the NPs [4]. Thus, a principal challenge in using SERS for sensitive and nondestructive detection is to localize the molecules of interest at the plasmonic surface but at a proper distance (2–4 nm) [2,4]. It is a common practice to demonstrate the SERS effectivity of a SERS-active substrate by using analytes that are strongly adsorbed at the plasmonic metal surfaces and located at the “hot spots”. The detection of analytes that are not interacting with (or adsorbing to) plasmonic surfaces remains an important practical task. This problem significantly hampers a wider practical application of SERS because even optimally fabricated SERS substrates lose their effectiveness when the analyte molecules cannot access the “hot spots”. To address this problem, various techniques have been developed to capture non-adsorbing molecules on SERS-active surfaces [3,18–20].

Among various plasmonic materials, silver SERS substrates provide the strongest Raman enhancement for the same structure [8] and are therefore promising for wide practical application. At the same time, Ag is much more chemically active than the widely used Au. Consequently, the use of the corresponding Ag-based SERS-active systems is complicated by additional chemical processes. The most exciting example is the influence of halide ions on the SERS signal. It is due to the high binding energy of Ag with halides, which is also responsible for the low solubility of Ag halides in water. The SERS activation of cationic analytes was observed in a number of studies after treatment of nanoparticles with halide ions [21–24]. The unusual effect that lithium chloride gives stronger SERS signal enhancement than other alkali metal halides was reported as well [23]. Also, the almost complete absence of the SERS signal of positively charged and neutral analytes (crystal violet and 9-aminoacridine) was reported for nanomaterials that were prepared in the absence of halides [22].

It should be noted that the role of halide ions in the SERS activation of Ag-based substrates is not yet fully clarified. A possible explanation could be their effect on the electrostatic interaction between analyte molecules and the surface of the Ag NPs. Literature analysis reveals that many authors use cationic organic dyes as SERS probing analytes [18,25]. In contrast, practically interesting biological molecules are mostly negatively charged. In 2015, the authors of [18] pointed out a possibility to prepare positively charged Ag NPs to analyze anionic analytes, which are hardly detectable by the Ag NPs prepared by common protocols. They used thiocholine to create a strong positive charge on the Ag NP surface. However, the solution of the charge problem was not so simple because citrate ions, which are used in many common protocols, caused aggregation of the resulting positively charged Ag NPs. Nevertheless, the proposed approach enabled the successful analysis of a number of negatively charged analytes. Silver NPs treated with polyethylenimine, spermine, or spermidine were successfully used for the detection of negatively charged oligonucleotides [19]. Finally, recent work [20] demonstrates the detection of anionic analytes by addition of multicharged metal cations. The abovementioned papers devoted to the chemical SERS activation of silver-based substrates, with the exception of [23–24], described colloidal NPs. The work [20] reports the absence of NP aggregation in the presence of multicharged metal ions. Considering the strong tendency of multicharged ions to destabilize colloidal systems [26–28] even at low concentrations, this observation is surprising. It should be noted that it is difficult to control the aggregation of NPs in colloidal substrates, which can occur even in the absence of any additives. SERS signals from aggregates due to the creation of “hot spots” can be orders of magnitude higher than the signal from the non-aggregated NPs [2,4]. Thus, even a small amount of the aggregates that is not visible in the absorption spectra of the solution can lead to a significant increase of the SERS intensity, obscuring the effect of functionalization of the NP surfaces. In contrast, solid substrates exhibit improved reproducibility of the SERS enhancement and provide the exact location of the target molecules, which makes it possible to exploit different techniques of functionalization.

To avoid different effects related to the aggregation of NPs we used Ag NPs immobilized by adsorption as a convenient and reproducible SERS substrate for the investigation of the chemical treatment of the Ag surface with different reagents. To study the electrostatic interaction with an analyte and exclude factors caused by complex specific interactions with the Ag surface, we used a set of organic thiols with differently charged functional groups and similar linker length. Additionally, we checked previously reported approaches on the surface modification of Ag (polymers, lithium chloride, and multicharged metal ions) to improve the SERS signal of substrates (our preliminary results have been described in [29]). Finally, we applied the obtained results to detect oligonucleotide molecules and showed that the addition of Cu^2+^ ions into the analyte leads to a good SERS signal.

## Results and Discussion

### Characterization of silver nanostructures

We synthesized Ag NPs with a size appropriate for SERS investigations (30–35 nm) with a relatively high NP concentration (32 µg/mL) sufficient for good immobilization. Size and shape of the NPs were analyzed from SEM and TEM images. To achieve a better resolution during SEM, we utilized a conductive silicon support and excluded the metal coating of the sample; Ag NPs were placed on the silicon surface by adsorptive immobilization to avoid aggregation during solvent evaporation.

**Figure 1:**
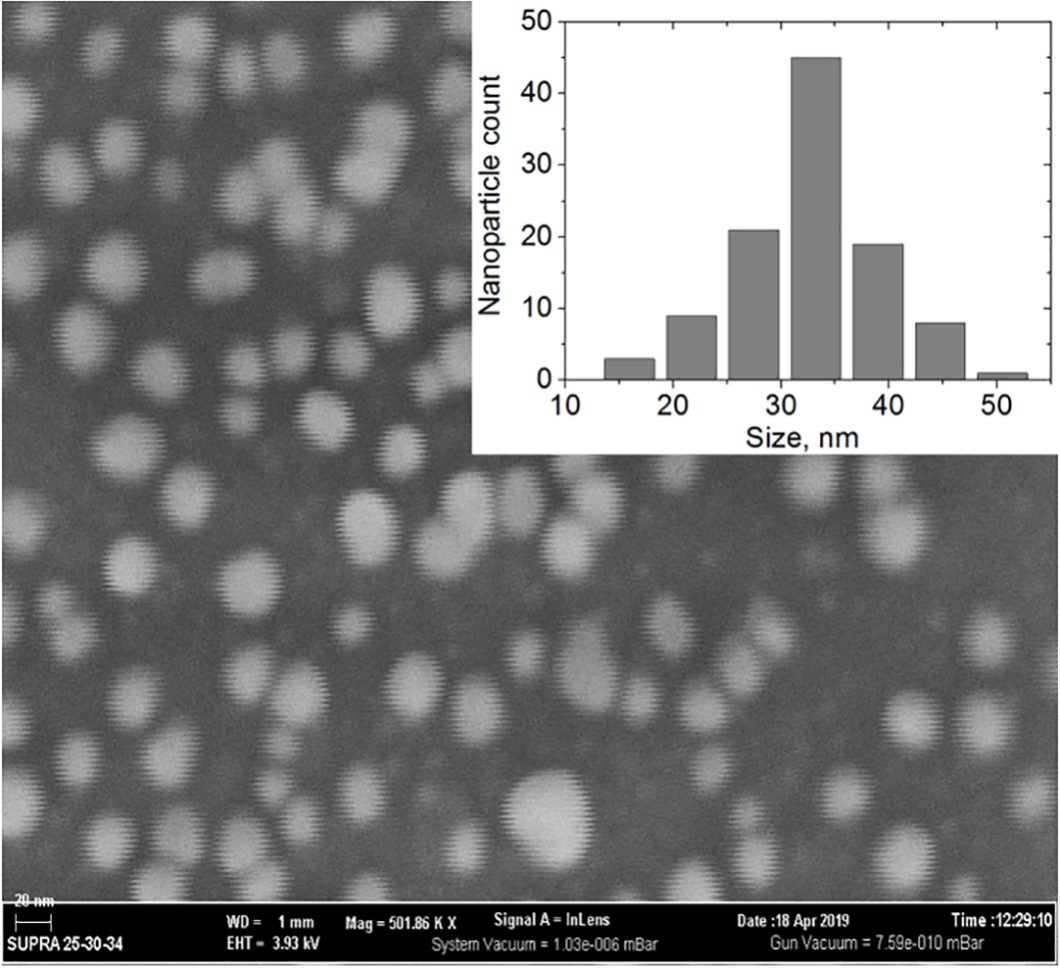
SEM image of Ag NPs and the corresponding size distribution histogram. For SEM imaging Ag NPs were deposited onto a PEI-modified silicon conductive support by adsorptive immobilization.

Figure 1 shows an SEM image of the NPs as well as the corresponding size distribution histogram. A similar TEM image (Figure S1, Supporting Information File 1) shows fewer nanoparticles and is therefore less reliable. Both methods give NP diameters in the interval of 30–35 nm. According to the SEM image, the NPs are mostly spherical with an average diameter of 33 nm. SEM and TEM images demonstrate a similar size distribution. Hence, we conclude that no NP size selection occurs during their adsorptive immobilization. The absorption spectrum of the Ag NPs (Figure S2, Supporting Information File 1) is in good agreement with electron microscopy and literature [30].

### Immobilization of Ag NPs on a glass surface

Utilizing glass slides for the preparation of SERS substrates permitted us to use siloxane chemistry to modify the surface and to control the immobilization process via the absorption spectra. Glass also minimizes additional effects related to SERS enhancement due to energy transfer to the support, which might occur when conductive or semi-conductive materials are used [31]. The immobilization of Ag NPs from sodium citrate solution was possible due to the poor adsorption of citrate ions on the Ag surface, which leads to a high immobilization efficiency. The sodium citrate concentration (2.5 mM) was adjusted to obtain a maximal NP surface filling and, at the same time, to avoid NP aggregation caused by high salt concentration.

**Figure 2:**
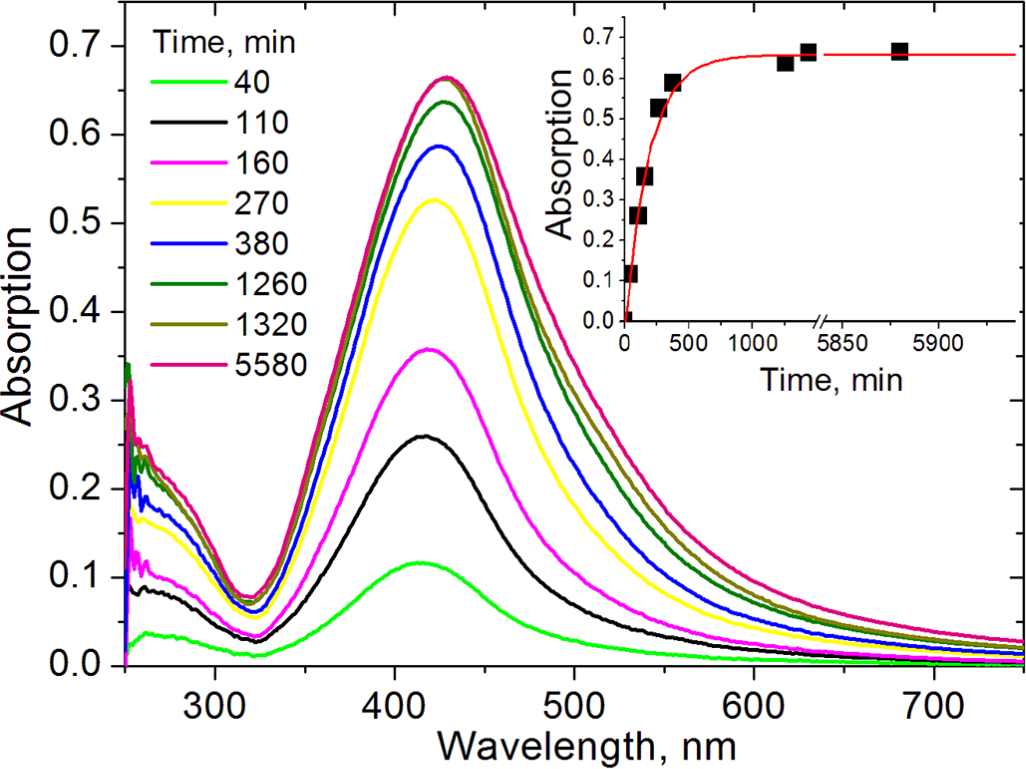
Immobilization kinetics of Ag NPs on a glass substrate. Since the glass slides were modified on both sides, the spectra correspond to the absorption of two NPs layers.

Figure 2 shows absorption spectra of the polyethylenimine (PEI)-modified glass support (slide) depending on the time of contact with Ag NPs. The maximum absorption value stabilizes after approximately 12 h with a shift from 412 to 427 nm. We suppose that the redshift of the absorption maximum is due to the plasmon interactions of the closely packed NPs.

**Figure 3:**
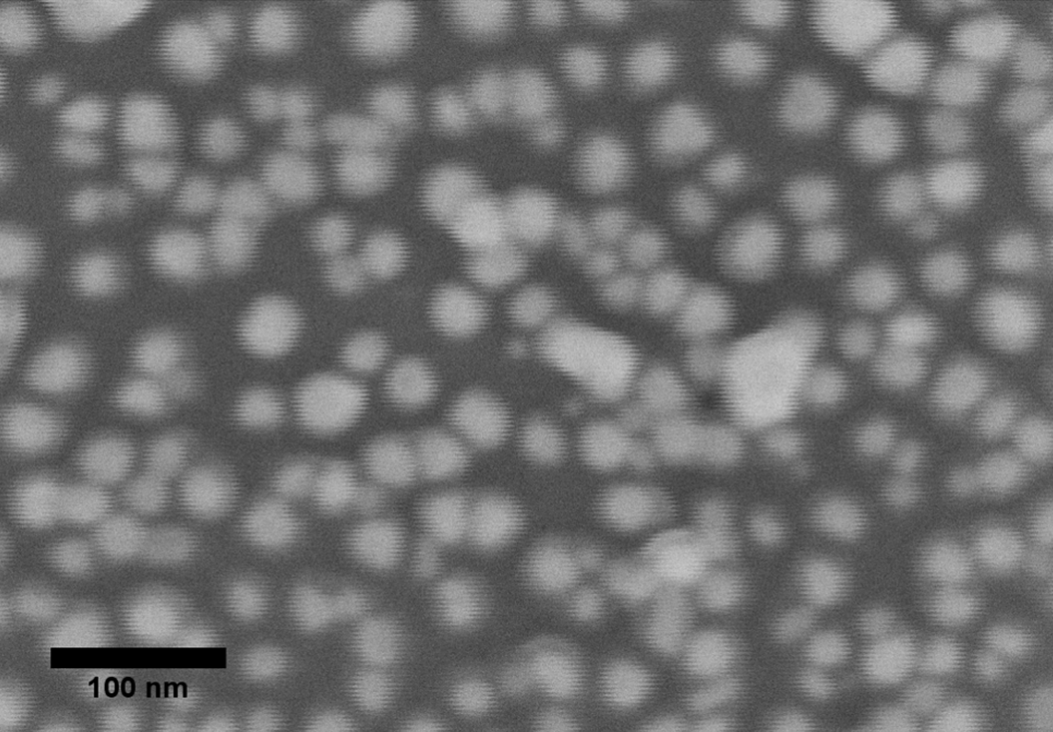
SEM image of Ag NPs immobilized on silicon surface by optimized procedure. The surface coverage degree is 58%.

In order to investigate the surface coverage of the glass SERS substrates, we used SEM images of the PEI-modified silicon support with analogous NP treatment supposing equal packing density (Figure 3). The surface coverage degree obtained was 58%, that is, 74% of the theoretical value for spherical particles with equal sizes and corresponds to the highest reported values for this method [10,12,32]. Moreover, the SEM image demonstrates many interparticle contacts, which are potential “hot spots” in SERS analysis.

### Electrostatic effect on SERS signal for thiol-modified Ag NPs

Despite the high NP packing density, about 42% of the support surface remains unoccupied, thus providing space for localization of the analyte in the case of poor interaction with the NP surfaces. Besides, a rough theoretical assumption of the thickness of the PEI modifying layer considering its molecular weight (25 kDa) and its branched structure gives a value below 10 nm. Taking into account the diameter of the NPs, we conclude that the major part of their surface would remain uncovered by the polymer layer in the plasmonic film on the SERS substrate. AFM images (Figure S3, Supporting Information File 1) confirm our conclusion. Topology map shows that the NPs are sticking out of the polymer layer. The adhesion image proves that the surface properties of the immobilized Ag NPs strongly differ from those on the glass substrate and that the polymer does not cover them. Weak citrate interaction with the Ag surface enables one to treat the NPs with different reagents and to study the influence of resulting surface effects on the SERS intensity. Thus, the prepared SERS substrates are a convenient instrument for surface affinity investigations with different surface modifications and analyte types.

**Figure 4:**
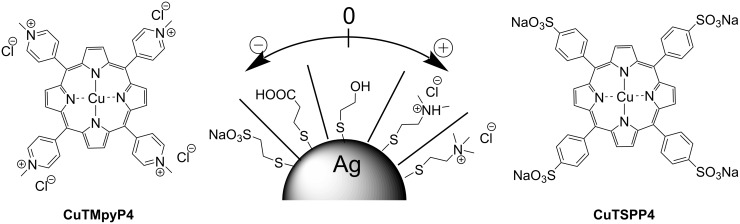
Schematic representation of the surface modification of immobilized Ag NPs by thiols bearing differently charged functional groups, and the oppositely charged model analytes CuTMpyP4 and CuTSPP4.

To exclude uncontrollable interactions of the analytes with the Ag surface, we used a set of organic thiols with the same linker length but differently charged hydrophilic functional groups. Thiol functionality guarantees a strong binding to the surface and a sufficiently dense molecule packing to make the surface properties determined by the introduced functional group. We used a short ethylene linker and hydrophilic functional groups to avoid lipophilic interactions. Two hydrophilic porphyrins with similar structures, equal charge value and opposite charge (CuTMpyP4 and CuTSPP4) were used as model analytes. Structures of the modifying molecules and the analytes are depicted in Figure 4.

**Figure 5:**
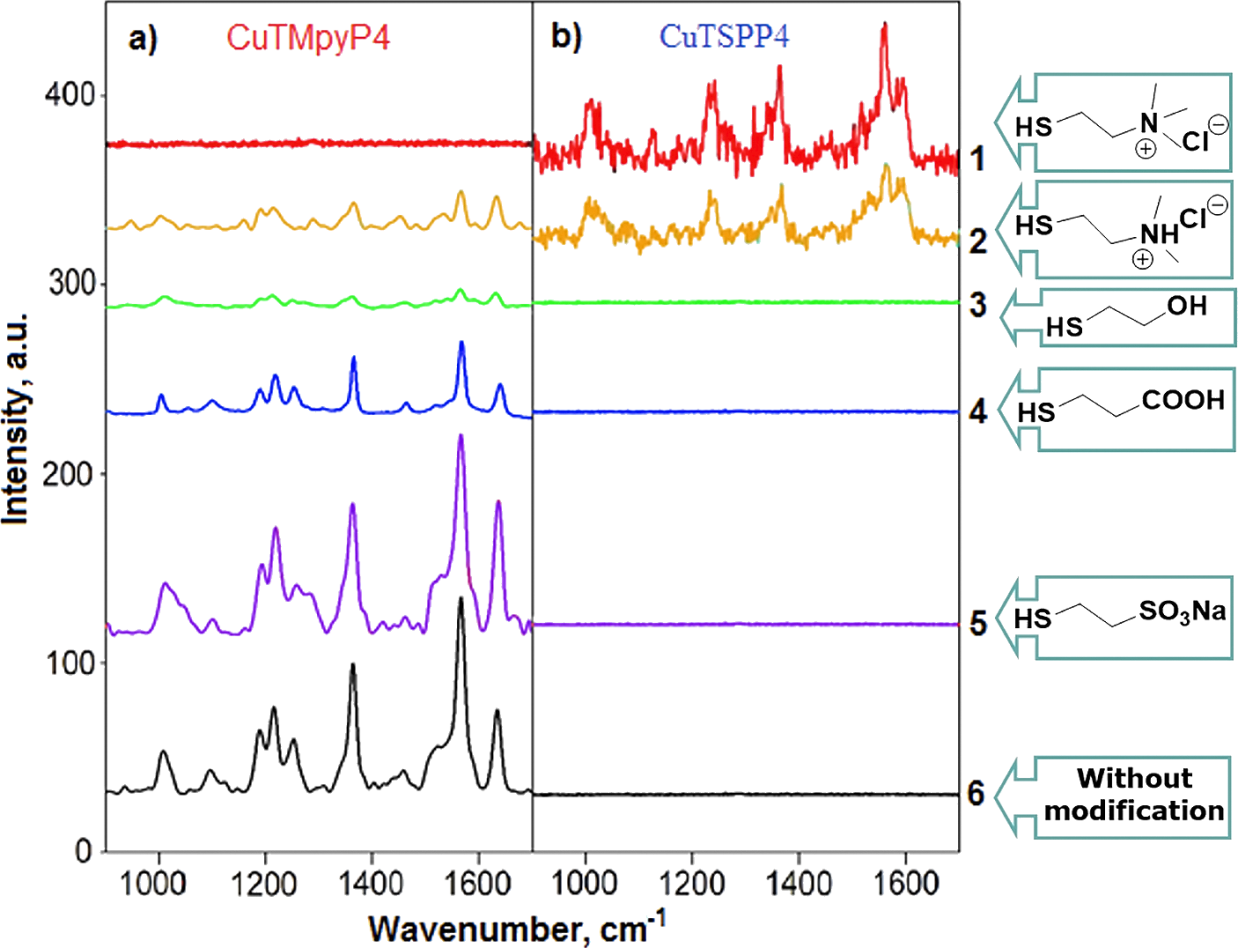
SERS spectra of (a) CuTMpyP4 and (b) CuTSPP4 drop-cast from 10^−6^ M solution onto the substrate modified with (1) thiocholine, (2) 2-(dimethylamino)ethanethiol hydrochloride, (3) 2-mercaptoethanol, (4) mercaptopropionic acid, (5) sodium mercaptoethyl sulfonate, and onto (6) the unmodified plasmonic surface.

The initially prepared plasmonic nanostructures yielded a rather intensive SERS signal for CuTMpyP4 bearing positive charge (Figure 5a, number 6). However, no spectrum was obtained for the negatively charged porphyrin CuTSPP4 (Figure 5b, number 6). Treatment of Ag NPs with sodium mercaptoethyl sulfonate did not result in a significant change of the SERS intensity for both porphyrins (Figure 5, number 5).

These data are in line with the initial negative charge of the NP surfaces. The high affinity of thiol groups to the Ag surface suggests that the chloride counter ions of CuTMpyP4 do not significantly influence the SERS signal. The use of mercaptopropionic acid for the modification leads to a decrease of SERS intensity of CuTMpyP4 (Figure 5, number 4). There is no SERS signal of CuTSPP4, presumably, because of the lowering of the negative charge of the Ag surface due to the several orders of magnitude lower acidity constant of the introduced carboxylic groups, compared to sulfate groups. Using neutral mercaptoethanol for surface modification led to an almost complete absence of SERS spectra for both porphyrins (Figure 5, number 3). In this case, the Ag surface became neutral and the analyte molecules were localized only on the glass support. Modification of the Ag NPs with 2-(dimethylamino)ethanethiol hydrochloride results in a SERS intensity decrease for the cationic analyte down to about a fourth. A weak CuTSPP4 spectrum is measured using this modification (Figure 5, number 2). This result proves a sufficient charge reduction due to the introduction of the weakly basic amine groups to the Ag surface. The presence of a SERS signal for both analytes can be explained by additional donor–acceptor interactions between the Cu atom and electronic pairs of the amine groups. Another explanation is the formation of zwitter-ionic structures on the NP surfaces. Thiocholine surface modification causes the complete disappearance of the CuTMpyP4 SERS signal and a significant spectrum intensity increase for CuTSPP4 (Figure 5, number 1). We explain this by positive charging of the Ag surface. However, the SERS signal of CuTSPP4 is much weaker in that case than that of CuTMpyP4 after negatively charged surface modification. This is presumably not caused by different adsorption maxima of the analytes because they have very similar optical properties [33]. We suggest that some negatively charged centers are still present at the NP surfaces after thiocholine modification, such as, for example, adsorbed chloride thiocholine counter ions, which contribute to the resulting surface charge. The experimental data obtained support previous reports about the great importance of the electrostatic interaction for charged analytes in SERS analysis [18,20] and once more emphasize the problem of SERS substrates with high surface charge.

### Other methods of silver surface modification

**Figure 6:**
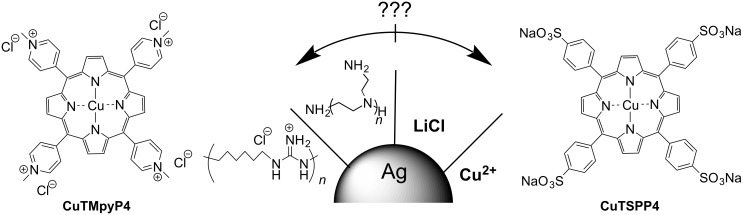
Schematic illustration of Ag surface modifications by PHMG, PEI, LiCl, and Cu^2+^, and charged model analytes CuTMpyP4 and CuTSPP4.

**Figure 7:**
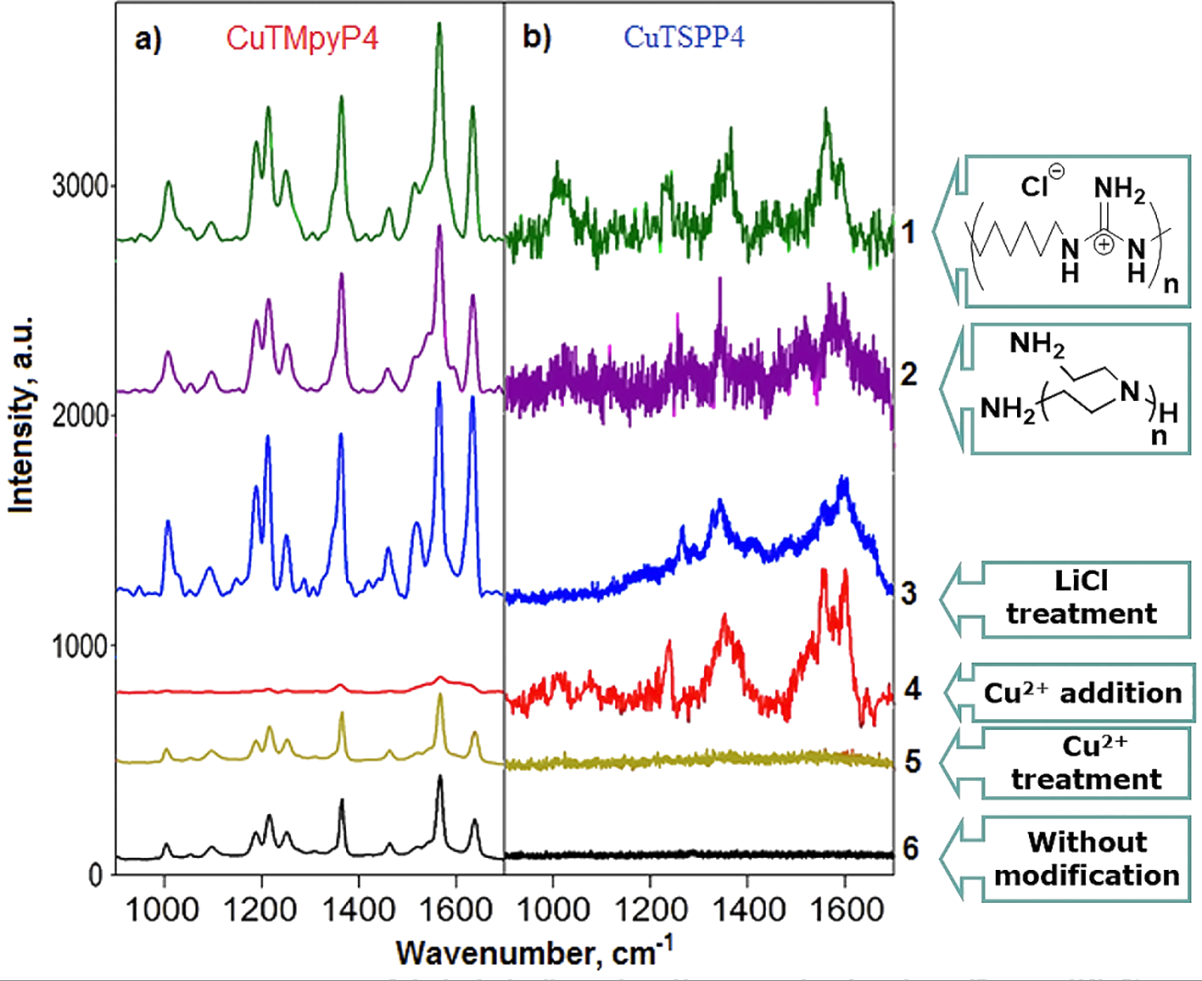
SERS spectra of (a) CuTMpyP4 and (b) CuTSPP4 drop-cast from 10^−6^ M solution onto the SERS substrate modified with (1) PHMG, (2) PEI, (3) LiCl, (4) Cu^2+^ (added into analyte solution), (5) Cu^2+^ (surface treatment), and onto (6) the unmodified plasmonic surface.

We also made experiments with other methods of plasmonic surface modification using our model SERS system, and compared the results with those reported in literature, for example, coating with polymers [7,19] or different inorganic reagents [20,22–23]. Figure 6 shows the corresponding modification schemes. Treatment of the plasmonic surface with lithium chloride led to a threefold increase of SERS intensity for the positively charged porphyrin (Figure 7a, number 3) whereas no spectra of anionic CuTSPP4 were observed. The result supports an earlier report about SERS activation by LiCl [23] and contradicts the authors’ statement that the effect does not depend on the surface charge. We suppose that the advantage of LiCl compared to other metal halides is due to poorly soluble LiAgCl_2_ formed at the Ag surface, which, according to our results, enhances the negative surface charge. However, the complex substance can form differently terminated regions on the surface, which can adsorb different molecules and give rise to their SERS signal. High specific affinity of Ag to the analytes could also promote SERS activity enhancement for the anionic analytes described in [23].

We also studied the SERS activity increase for negatively charged analytes using multicharged cations [20]. For this purpose, copper cations were selected since they are already present in the structure of both analyte molecules and, thus, their chemical transformation can be excluded. Treatment of the NP surfaces with Cu^2+^ ions resulted in an about twofold increase of the CuTMpyP4 SERS intensity. Under the same conditions, the CuTSPP4 SERS spectrum was absent (Figure 7, number 5). We suggest that Cu^2+^ ions are weakly adsorbed at the Ag surface and were mostly removed during the washing stage. Copper complexation with PEI and positive charging of the glass surface is expected in this case. This could also explain the SERS increase of the cationic analyte. Furthermore, we checked the effect of adding Cu^2+^ cations to the analyte on the SERS intensity. The SERS intensity of CuTMpyP4 decreases to a fifth in the presence of Cu^2+^ ions (Figure 7, number 4). At the same time, a weak SERS signal of CuTSPP4 was detectable. These observations suggest that the charge of the NP surfaces remains negative and Cu^2+^ ions do not create a strong positive charge on the Ag surface. We suppose that CuTSPP4 detection in this case is related only to the shielding of the analyte molecules by Cu^2+^ ions.

Polyethylenimine and polyhexamethylene guanidine (PHMG) were used as basic polymer modifiers to create positive charges on the Ag surface (Figure 7, numbers 1 and 2). Both of them were described as effective Ag NP stabilizers, which lead to NPs with positive zeta potential [7,19]. We found that treatment of the Ag NPs with those polymers resulted in a significant increase of the CuTMpyP4 SERS signal, that is, threefold for PEI and fourfold for PHMG. A weak CuTSPP4 spectrum was only observable in the case of PHMG. We suppose that the results are caused by a partial desorption of polymer molecules from the surface by washing. The remaining polymer molecules are not able to generate a strong positive charge at the surface but they improve the adsorption of the analyte molecules due to hydrogen bonding and donor–acceptor and lipophilic interactions.

### SERS analysis of dye-labeled oligonucleotides

Biomolecules such as nucleotides and nucleic acids are of great interest for practical SERS applications. Oligonucleotides are the most suitable biomolecules for electrostatic interaction studies because of their strong negative charge. Recently, we showed the crucial role of electrostatic interactions of oligonucleotides with NP surfaces for bioconjugation [34]. Thus, oligonucleotide SERS spectra can indicate a positive charge of the SERS substrate surface. An oligonucleotide was labeled with a Black Hole Quencher dye (BHQ1) for better spectrum recognition due to exclusion of characteristic peaks with background fluorescence, which is possible in the case of luminescent dyes. As BHQ1 has a wide band that overlaps the excitation laser frequency, we can speak about surface-enhanced Raman resonance scattering (SERRS) in this case.

**Figure 8:**
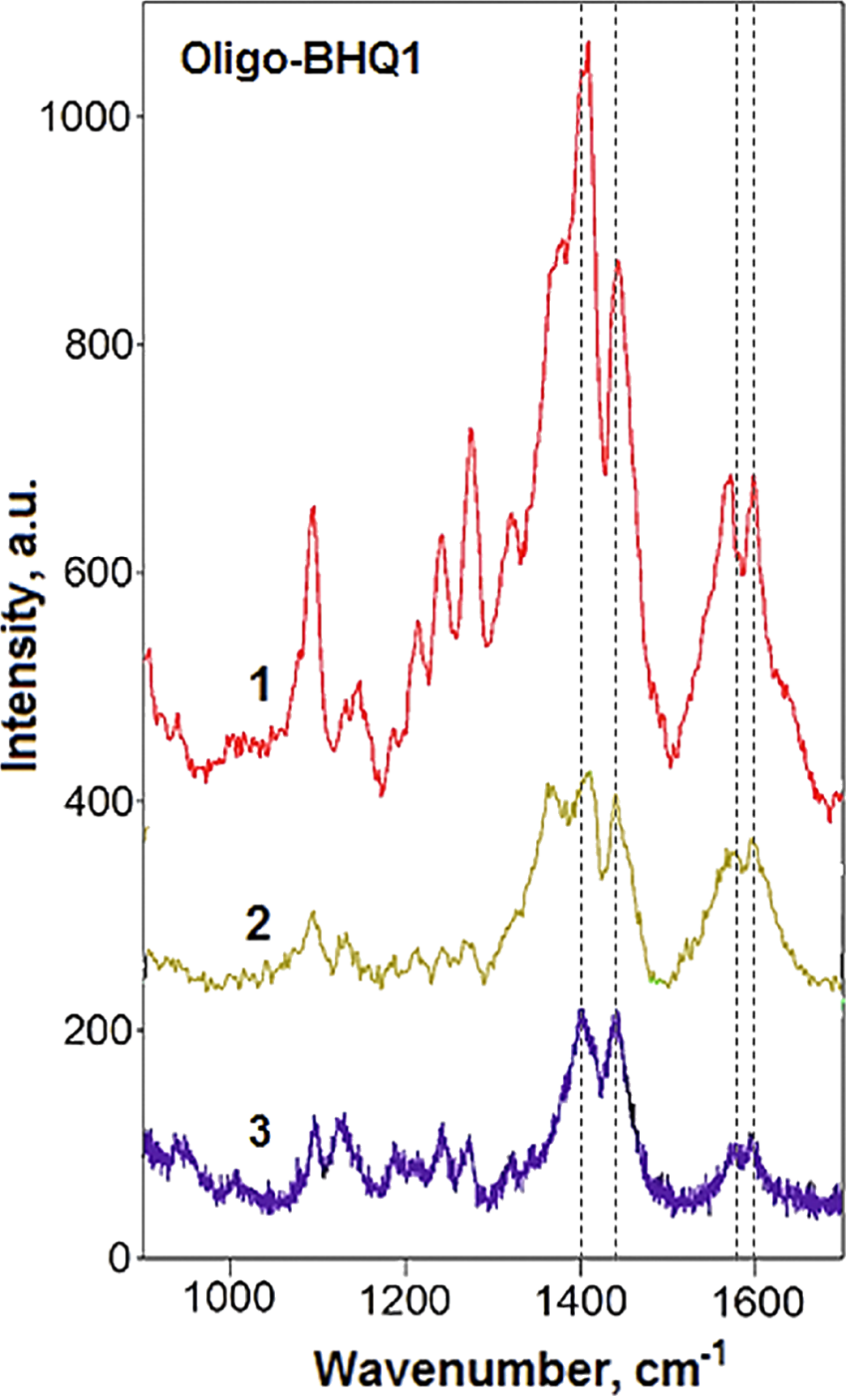
SERS spectra of BHQ1-labeled oligonucleotide drop-cast from 10^−5^ M solution onto substrates modified with (1) Cu^2+^ (added into the analyte solution) and (2) Cu^2+^ (surface treatment); (3) Raman spectrum of BHQ1 in Ag NP solution.

The unmodified SERS substrate did not show any SERS signal of the oligonucleotide (Figure 8). Based on our previous experiments on Ag surface modification with thiols, we achieved the best results for the negatively charged analytes by using thiocholine as a modifier. The method enabled the measurement of a sufficiently good CuTSPP4 spectrum. We applied this approach to obtain the SERS spectrum of the oligonucleotide but failed. This supports our idea about negatively charged centers at the Ag surface that decrease the resulting positive charge of the thiocholine-modified surface. Consequently, the PEI-modified glass surface becomes more suitable for the competitive oligonucleotide localization.

From a range of different modifications, only treatment with copper ions appeared to be successful (Figure 8, numbers 1 and 2). According to an earlier investigation [35], the Cu^2+^ ions are able to bind effectively both the phosphate groups and the nucleobases in the oligonucleotide molecules to yield charge inversion. This is a key factor for the oligonucleotide detection with Cu^2+^-modified SERS substrates. The spectrum detection after Cu^2+^ treatment, followed by washing (which was not working for CuTSPP4), supports that and can be explained by a low Cu^2+^ ion concentration created by a residual PEI–copper complex. Additionally, it was confirmed in experiments with Mg^2+^ ions, instead of Cu^2+^ ions, which are much less capable of complexation with nucleobases. Moreover, Mg^2+^ cations have been shown [35] to have the lowest affinity to DNA molecules among doubly charged ions (Mg^2+^ < Co^2+^ < Ni^2+^ < Mn^2+^ < Zn^2+^ < Cd^2+^ < Cu^2+^) and, in contrast to the other ions, they do not induce unwinding of the double helix. Addition of Mg^2+^ ions to the analyte did not yield measurable SERS spectra of oligonucleotides or CuTSPP4.

Thus, our results emphasize the importance of the SERS substrate surface charge for successful SERS detection and point out another possible way to control it, that is, through analyte charge inversion by interaction with positively charged metal cations. This approach requires the selection of metal cations with high affinity to the analyte molecules. As demonstrated in [35], this selection is not always easily predictable from basic considerations regarding complexation.

## Conclusion

Effects of electrostatic interaction between analyte molecules and plasmonic surfaces on the SERS enhancement was investigated by using Ag NPs immobilized on a glass support. Two oppositely charged porphyrins with similar structure and the same formal charge (cationic CuTMpyP4 and anionic CuTSPP4) were used as model analytes. The surface charge of Ag NPs was adjusted by hydrophilic thiols. In the case of cationic CuTMpyP4, a shift from an initially negative Ag surface charge to a positive charge leads to a SERS intensity decrease up to complete spectrum disappearance. On the other hand, positive charging of the Ag surface enables the measurement of CuTSPP4 spectra, which is prevented for the surface modifications with negative charge. However, it remains relatively weak, revealing that the Ag surface has a tendency to be negatively charged. Thus, the generation of a strong positive charge on the Ag surface is important for successful SERS detection of negatively charged analytes.

Previously described approaches for Ag surface modification such as PEI, PHMG, LiCl, and multicharged metal ions led mainly to a SERS enhancement of CuTMpyP4, that is, threefold for PEI and fourfold for PHMG and LiCl. Only the addition of Cu^2+^ ions to the analyte enabled a satisfactory measurement of the CuTSPP4 SERS spectrum. Nevertheless, under similar conditions, the SERS signal of CuTMpyP4 was still present, which means that the surface charge was not inverted by the addition of Cu^2+^ ions. At the same time, this was the only method that allowed us to get a SERS spectrum of a negatively charged dye-labeled oligonucleotide, which was shown to interact strongly with Cu^2+^ ions. We suppose that the charge inversion of the analyte is the key factor in this case. Thus, our results shift the focus from the Ag surface modification to the charge inversion of the analyte molecules, showing its perspective use for negatively charged analytes.

## Experimental

### Materials

Dye-labeled oligonucleotide 5′-CCTGCGATCTCTCTATCCAG-[BHQ1]-3′ was purchased from Primetech ALC (Minsk, Belarus). Cu(II) tetrakis(4-*N*-methylpyridyl)porphyrin, tetrachloride salt (CuTMPyP4), and Cu(II)-5,10,15,20-tetrakis(4-sulfonatophenyl)porphyrin, tetrasodium salt (CuTSPP4), were purchased from Frontier Scientific (Logan, U.S.A.). Cellulose acetate membrane (cutoff = 12 kDa), branched polyethylenimine (PEI, *M*_W_ = 25000), polyhexamethylene guanidine hydrochloride (PHMG), analytical grade DMSO, NaI, LiCl, AgNO_3_, sodium mercaptoethyl sulfonate, mercaptopropionic acid, 2-mercaptoethanol, 2-(dimethylamino)ethanethiol hydrochloride, acetylthiocholine chloride, sodium citrate, and other reagents, if not mentioned otherwise, were purchased from Merck and used without additional purification.

### Instrumentation and Software

Absorption and extinction spectra of the samples were measured using PB 2201 spectrometers (SOLAR, Belarus). Scanning electronic microscopy (SEM) images were recorded using a Zeiss LEO SUPRA 25 (Germany). Transmitting electron microscopy (TEM) images were recorded using a Zeiss LEO 906E (Germany). SEM and TEM images were treated using ImageJ 1.51k freeware. AFM images were scanned in air using a BioScopeResolve (Bruker) atomic force microscope in PeakForceQNM mode with recording the adhesion force maps and topographic images. SERS measurements were carried out by using a scanning probe Raman microscope “NanoFlex” (Solar LS, Belarus). The source of excitation at 488.0 nm was an argon ion laser (Melles Griot, USA). Excitation and measurement of Raman scattering was carried out using a 100× objective and a CCD camera Newton 970 EMCCD DU970P-BV (Andor Technology Ltd, UK). Additionally, in some cases, the SERS spectra were recorded using a Raman spectrometer equipped with Spex 270M (Jobin Yvon) spectrograph and liquid-nitrogen-cooled CCD detector (Princeton Instruments). Spectra were excited by a 441.6 nm of a He–Cd laser.

### Synthesis of silver NPs

The synthetic procedure was analogous to that described in [36]. 50 mL of deionized water, 42 mg of sodium bicarbonate, 15 mg sodium citrate, and 450 mg of water-free glucose were added into a round bottom flask and stirred until complete dissolution. Then, 640 µL of 4 mg/mL silver nitrate solution were added under vigorous stirring and kept for 4 h in the ultrasonic bath. The resulting silver nanoparticle solution was dialyzed against 2.5 mM sodium citrate and stored at 4 °С.

### Glass and silicon surface functionalization

Standard optical microscope glass slides of 1.0 mm thickness were used as base substrates. As a silicon support, 5 mm square-shaped polished chips of Sb-doped electrically conductive silicon were used. The modification was performed in a similar way as described in [36]. The same modification protocol was applied as for glass and the silicon supports. The substrates were treated with a 1:1 (by mass) mixture of concentrated H_2_SO_4_ and 30% hydrogen peroxide water solution for 2 h, rinsed with distilled water, and dried. The cleaned substrates were treated with a solution of (3-chloropropyl)trichlorosilane in water-free toluene (2% by mass) for 24 h and rinsed several times with dry toluene, then with isopropyl alcohol, and dried at room temperature. The chloropropyl-functionalized substrates were then modified with PEI by soaking in 10% (by mass) polymer solution in DMSO at 80 °C for 5 h in the presence of catalytic quantities of sodium iodide. Finally, the substrates were repeatedly rinsed with hot distilled water and dried.

### Silver NPs adsorptive immobilization kinetics study

PEI-modified glass substrates 5 × 10 mm in size were placed in 3 mL (great excess) of Ag NP water solution (Ag concentration of 32 µg/mL) with 2.5 mM sodium citrate and kept at room temperature. A 2.5 mm quartz cuvette filled with 2.5 mM sodium citrate water solution was used to measure the absorption spectra. The glass substrate covered by Ag NPs was placed into the cuvette after washing with citrate buffer. The spectrum was immediately measured, and the substrate was returned into the Ag NPs solution. The absorption spectra were obtained against the analogous blank glass substrate.

### Chemical modification of the plasmonic surface

PEI-modified glass substrates were kept in Ag NP water solution (silver concentration of 32 µg/mL) with 2.5 mM sodium citrate at room temperature for 24 h. The resulting substrates were washed with deionized water and further chemically treated or dried for use without additional modification. The same protocol was applied for the preparation of silicon substrates for SEM images.

The glass-based SERS substrates were treated with 0.1 mg/mL solutions of a series of organic reagents (sodium mercaptoethyl sulfonate, mercaptopropionic acid, 2-mercaptoethanol, 2-(dimethylamino)ethanethiol hydrochloride, acetylthiocholine chloride, PEI, and PHMG). Thiocholine was generated in situ by basic hydrolysis of its acetyl ether in sodium carbonate buffer at pH 10.8. The supports were soaked for 30 min in the corresponding reagent solution, washed with deionized water, and dried. Treatment with doubly charged metal ions was carried out in 5 mg/mL Cu sulfate or Mg sulfate solution for 30 min with subsequent washing and drying. Lithium chloride treatment was performed according to [23].

### Preparation of samples for SERS analysis

To assess the SERS substrate activity, water solutions of cationic CuTMPyP4 (1 µM), anionic CuTSPP4 (1 µM), or oligonucleotide (10 µM) were used as a probe analyte. The solutions were drop-cast onto the substrate (ca. 100 µL/cm^2^), and dried at room temperature. In the case of metal ion addition, 5 mM of the corresponding sulfate was added to the analyte solution.

### SERS acquisitions parameters

The SERS spectra were obtained in back-scattering geometry through the microscope objective. The estimated diameter of the laser spot focused on the substrate was 1–2 µm. SERS spectra for every sample were measured at 5–10 different points on its surface. Each spectrum was accumulated for 5 s. The SERS intensity distribution on the substrate surface was rather uniform, that is, the deviation between different point was less than 10% for each sample. An average representative graph has been chosen for demonstration in Figures 5, 7, and 8).

## Supporting Information

File 1 Characterization of silver nanostructures.
