# Supplementary material for: Modification of a SERS-active Ag surface to promote adsorption of charged analytes: effect of Cu2+ ions
Source: Beilstein J Nanotechnol. 2021 Aug 16;12:902–12. doi: 10.3762/bjnano.12.67 (PMC8381809; doi:10.3762/bjnano.12.67)
Supplement: File 1 — Characterization of silver nanostructures. [file Beilstein_J_Nanotechnol-12-902-s001.pdf]

BEILSTEIN JOURNAL OF NANOTECHNOLOGY

Supporting Information

for

Modification of a SERS-active Ag surface to promote adsorption of charged analytes: effect of Cu^{2+} ions

Bahdan V. Ranishenka, Andrei Yu. Panarin, Irina A. Chelnokova, Sergei N. Terekhov, Peter Mojzes and Vadim V. Shmanai

Beilstein J. Nanotechnol. **2021,** *12,* 902–912. doi:10.3762/bjnano.12.67

Characterization of silver nanostructures

License and Terms: This is a supporting information file under the terms of the Creative Commons Attribution License (https://creativecommons.org/licenses/by/4.0). Please note that the reuse, redistribution and reproduction in particular requires that the author(s) and source are credited and that individual graphics may be subject to special legal provisions.
The license is subject to the *Beilstein Journal of Nanotechnology* terms and conditions: (https://www.beilstein-journals.org/bjnano/terms)

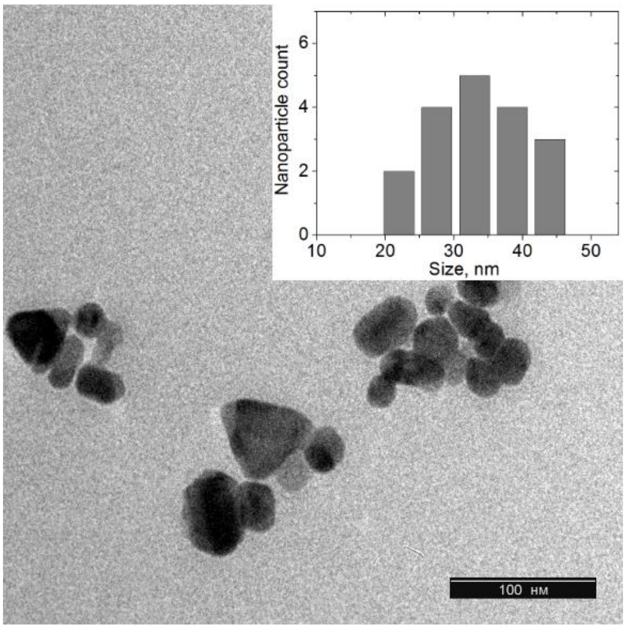

Figure S1: TEM image of silver nanoparticles and corresponding size distribution histogram. The TEM sample was prepared by simple drop-casting.

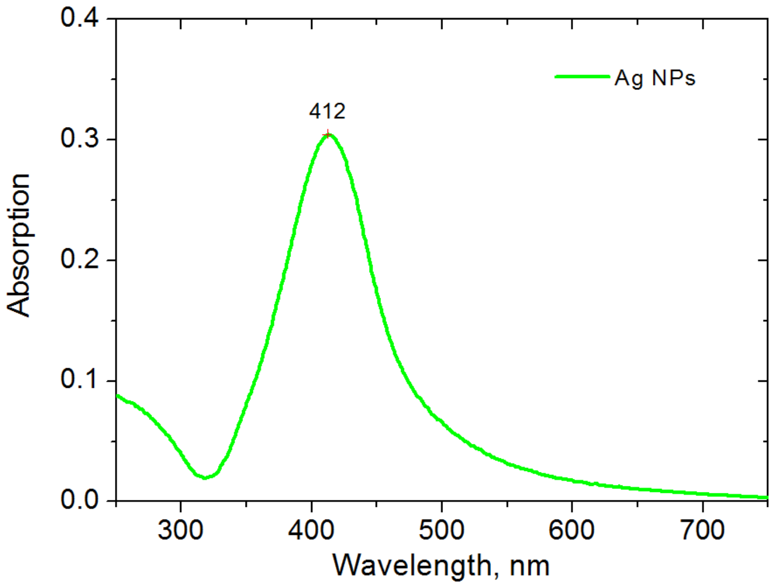

Figure S2: Absorption spectrum of the colloidal silver NPs.

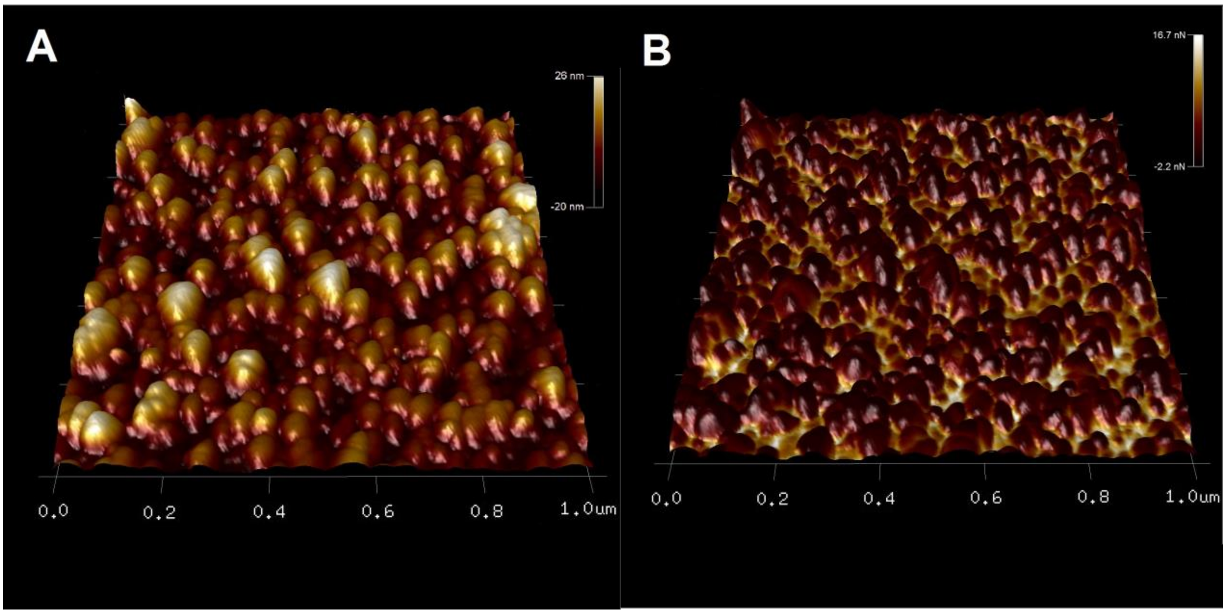

Figure S3: AFM images of silver NPs immobilized on a PEI-modified silicon support. A) Topological map; B) adhesive properties map (scan size of 1 μm × 1 μm; scan rate of 0.5 Hz (256 × 256 pixels); peak force setpoint of 0.5 nN).
